# The role and challenges of intratumoral microbiota in colorectal cancer immunotherapy

**DOI:** 10.3389/fphar.2025.1634703

**Published:** 2025-08-07

**Authors:** Miao Hao, Haiming Xu, Min Li, Dan Jiao

**Affiliations:** ^1^ Scientific Research Center, China-Japan Union Hospital of Jilin University, Changchun, China; ^2^ Department of Cardiology, China-Japan Union Hospital of Jilin University, Changchun, China; ^3^ Department of Respiratory and Critical Care Medicine, China-Japan Union Hospital of Jilin University, Changchun, China; ^4^ Department of Ultrasound, China-Japan Union Hospital of Jilin University, Changchun, China

**Keywords:** colorectal cancer, intratumoral microbiota, tumor microenvironment, tumor therapy, chemotherapy, immunotherapy

## Abstract

Colorectal cancer (CRC) is the third most common malignant tumor globally, and its development is closely related to interactions between the host and microbes. Recent studies have shown that the diversity of intratumoral microbiota significantly influences CRC progression and responses to immune therapy. This influence occurs through mechanisms such as immune microenvironment regulation, metabolic reprogramming, and epigenetic modifications. However, there is still a lack of systematic analysis regarding the diversity of intratumoral microbiota in CRC and its immune regulatory mechanisms, particularly in the metabolic and immune regulation. This article presents a systematic review of the compositional characteristics of intratumoral microbiota in CRC, the associated immune regulatory mechanisms, and their roles in chemotherapy and immunotherapy. It also discusses challenges like standardizing microbiome detection methods and the ethics of clinical translation, while proposing a strategy for integrating multi-omics using artificial intelligence. This article provides a theoretical basis for developing personalized treatment regimens that target the microbiota.

## 1 Introduction

Colorectal cancer (CRC) is one of the leading causes of cancer-related deaths worldwide. Epidemiological data indicate that CRC has become the third most common cancer worldwide, influenced by complex interactions among diet, lifestyle, and genetic factors (Sung et al., 2021). As traditional treatments face resistance challenges, tumor immunotherapy has brought new hope to advanced CRC patients by activating host anti-tumor immune responses. However, clinical data reveal that only 12%–15% of CRC patients have sustained responses to PD-1/PD-L1 inhibitors. This significant variability among patients is closely related to the heterogeneous immune environment within tumors (Cai et al., 2024).

Recent studies have identified distinct microbial communities in CRC tumor tissues that differ significantly in composition from the gut’s symbiotic microbiota (Nejman et al., 2020; Zhou P. et al., 2022). Intratumoral microbiota primarily colonize through three pathways: direct migration by disrupting the intestinal barrie, hematogenous invasion via the circulatory system, and migration from adjacent tissues (Cao et al., 2024). These colonizing microbiota significantly affect the immunogenicity of tumor cells through mechanisms including epigenetic regulation, secretion of metabolic products, and activation of pattern recognition receptors (Li et al., 2023). This finding offers a new perspective on how microbiota contribute to resistance against immunotherapy. Nevertheless, research on the microbiota-immune interaction network in CRC remains limited, particularly regarding metabolic-immune cross-regulation.

In assessing microbial community complexity, alpha and beta diversity serve as foundational metrics (Wei et al., 2025). Alpha diversity quantifies species richness and evenness within a single sample, commonly measured by: Shannon Index (reflecting species abundance and uniformity), Simpson Index (emphasizing dominance patterns), and Chao1 Index (estimating total species richness from rare taxa counts) (Maley et al., 2024). Conversely, beta diversity evaluates compositional differences across samples using distance-based metrics: Bray-Curtis Dissimilarity (comparing abundance profiles) and Weighted UniFrac Distance (incorporating phylogenetic relationships) (Kim et al., 2023; Lozupone and Knight, 2005). In CRC tumors, these indices map spatial heterogeneity—alpha diversity revealing niche-specific microbial load, while beta diversity distinguishes intratumoral sub-regions and correlates with molecular subtypes (Byrd et al., 2025; Zhao et al., 2021). This analytical framework is critical for decoding microbial-driven immunotherapy resistance.

Current research has two main limitations: on one hand, most studies focus on the association between gut microbiota and CRC immunotherapy, leaving the independent mechanisms of intratumoral microbiota in CRC unclear. On the other hand, research on the cross-regulation of the CRC intratumoral microbiota-metabolism-immune axis is still in early stages, especially concerning how bacterial metabolites influence immune cells and cytokines in the tumor microenvironment (Gutiérrez-Salmerón et al., 2021; Wang J. et al., 2021). Furthermore, the diversity of intratumoral microbiota, including its spatial distribution, microbial interactions, and links to CRC molecular subtypes, needs further clarification (Gao et al., 2022).

An in-depth exploration of the relationship between intratumoral microbiota and tumor immunotherapy in colorectal cancer will help us understand how tumors evade the immune system and may also reveal new strategies to optimize immunotherapy. This review aims to discuss the dual roles of intratumoral microbiota in CRC immunotherapy and the challenges in clinical translation. By integrating cutting-edge evidence, this article aims to provide a theoretical basis for optimizing targeted strategies for CRC immunotherapy and to promote the clinical translation of microbiota-immune interaction research.

## 2 Colorectal cancer and its intratumoral microbiota

### 2.1 Characteristics of the intratumoral microbiota in colorectal cancer

#### 2.1.1 Detection technologies and methods for intratumoral microbiota in colorectal cancer

The primary detection technologies for intratumoral microbiota in CRC are 16S rRNA gene sequencing, metagenomic sequencing, and transcriptomic analysis. These technologies work together to reveal the composition, function, and interactions of microbial communities within the tumor microenvironment. 16S rRNA gene sequencing, the most widely used method for detecting intratumoral microbiota. It efficiently identifies microbes at the genus or species level by amplifying conserved 16S rRNA gene fragments, making it suitable for analyzing the diversity of large-scale samples (Chao 1 and Shannon index were used to estimate alpha diversity, beta diversity was used in QIIME) (Zwinsová et al., 2021). For example, this technology has been used to identify the microbial composition of 1,526 tumors and adjacent tissues from seven types of cancer (Nejman et al., 2020). Metagenomic sequencing offers detailed insights into microbial community structure and functional gene analysis, which are essential for understanding the role of microbes in tumor progression (Hardwick et al., 2018). Additionally, transcriptomic analysis can reveal interactions between microbes and host gene expression, further exploring the impact of microbes on the tumor microenvironment. Recently developed methods that combine single-cell metagenomics with spatial metabolomics enable the localization of microbial communities at the subcellar level (Xu and Zhao, 2018; Zhang et al., 2023). For instance, spatial transcriptomics and single-cell RNA sequencing have confirmed that *Fusobacterium* nucleatum in CRC is primarily enriched in CD45^+^ immune cells and CD66b+ myeloid cells (Galeano Niño et al., 2022). The combined application of these technologies provides technical support for comprehensively analyzing the composition, function, and spatial heterogeneity of intratumoral microbiota in CRC.

#### 2.1.2 Composition and characteristics of intratumoral microbiota in colorectal cancer

The intratumoral microbiota in CRC exhibits specific compositional characteristics closely related to tumor stage, molecular subtype, and prognosis. For example, *Fusobacterium* nucleatum is widely recognized as a marker for advanced CRC. Its abundance in CRC tissues is positively correlated with TNM staging (Ma et al., 2024; Naghavian et al., 2023). Furthermore, other bacterial phyla, such as *Bacteroides*, Firmicutes, and Proteobacteria, are also enriched in CRC (Gao et al., 2022; Xu et al., 2023; Zhou X. et al., 2022). Studies have shown that the composition of intratumoral microbiota is closely associated with the host’s immune status, tumor stage, and prognosis. This suggests that microbes may influence tumor occurrence and development by regulating the tumor microenvironment (Zhang H. et al., 2024).

#### 2.1.3 Clinical significance of heterogeneity in intratumoral microbiota in colorectal cancer

The diversity, abundance, and distribution of intratumoral microbiota in CRC are important for evaluating prognosis and treatment strategies. In CRC patients, the tumor microbiota usually exhibits higher diversity compared to that of healthy individuals (Zhang et al., 2025). For instance, the microbial alpha diversity index is lower in CRC tumor tissues than in adjacent tissues, and the differences in beta diversity are significantly linked to TNM staging (Long et al., 2024; Zhang et al., 2025). Studies indicate that intratumoral microbiota diversity is closely related to tumor prognosis, with lower microbial diversity usually associated with poorer prognosis (Jin et al., 2024; Zhang et al., 2025). A positive correlation exists between microbial diversity in CRC tumors and tumor diameter, following a power-law model. This suggests that larger tumors tend to have greater internal microbial diversity, indicating that intratumoral microbiota likely play a significant role in the tumor microenvironment and cancer progression (Dovrolis et al., 2024).

Bullman et al. used 16S rRNA gene sequencing combined with spatial transcriptomics to identify varied spatial distribution heterogeneity of bacterial communities such as *Fusobacterium* nucleatum, *Bacteroides*, and Leptotrichia in CRC tumor tissues (Galeano Niño et al., 2022). The abundance of Prevotella, *E. coli*, and Phascolarctobacterium significantly increased in CRC liver metastatic tissues (Gu et al., 2024). In CRC patients, the relative abundance of Alistipes and Blautia was positively correlated with survival probability. In contrast, in rectal cancer patients, the abundance of Porphyromonas was positively correlated with tumor stage (Wang L. et al., 2021).

Furthermore, studies have shown a significant relationship between the diversity of microbes in tumor tissues and the presence of tumor-infiltrating lymphocytes. This suggests that the composition of microbial communities may somewhat reflect the immune microenvironment of tumors (Bhatnagar et al., 2024). Longitudinal cohort studies indicate that antibiotic exposure can increase *Fusobacterium* levels in tumors while reducing CD8^+^ T cell infiltration. This implies that the stability of microbial community impacts immune surveillance (Popkes and Valenzano, 2020). Additionally, the stability of intratumoral microbiota is influenced by various factors, including diet, antibiotic use, and the host’s immune status (Bhatnagar et al., 2024). For example, certain probiotics, such as *Lactobacillus* rhamnosus GG, can enhance intestinal immune responses and promote the stability of microbial communities, which may potentially improving treatment outcomes for CRC patients (Owens et al., 2021). These findings provide valuable insights for improving early diagnosis and developing targeted therapies for CRC (Lee et al., 2021; Okuda et al., 2021) (Table 1).

**TABLE 1:** Comprehensive profiling of intratumoral microbiota in colorectal cancer: origins, methodologies, and clinical associations.

Time	Source	High expression	Low expression	Methods	Results	References
2022	CRC OSCC	*Fusobacterium* *Bacteroides* Leptotrichia Parvimonas Peptoniphilus	----	spatial-profiling technologies single-cell RNA sequencing INVADEseq	The intratumoral microbiota drives tumor heterogeneity by creating an immunosuppressive microenvironment and regulating the transcriptional programs of cancer cells	Farhadi Rad et al. (2024)
2024	Colorectal cancer liver metastasis	Prevotella *Escherichia coli*,*E. coli*,E.coli Phascolarctobacterium	----	16S rDNA	*E. coli* promotes the progression of colorectal cancer liver metastasis by enhancing lactic acid production and facilitating macrophage M2 polarization	Gubernatorova et al. (2023)
2024	CRC	Right hemicolon *Fusobacteria* 、 *Alistipes* Left hemicolon *Firmicutes* 、 *Bacteroidetes*	Bacteroidetes Firmicutes Verrucomicrobia Actinobacteria Euarchaeota		The microbial community in CRC exhibits site and molecular subtype-specific distribution, regulating tumor progression through multiple pathways	Gopalakrishnan et al. (2018)
2024	CRC treatment	----	----	Bird shotgun metagenomic sequencing	Orally administered hydrogel (Oxa@HMI) treats CRC by significantly increasing beneficial bacteria in the intestines and tumors while reducing pathogenic bacteria	Lozupone and Knight (2005)
2023	Locally advanced rectal cancer (LARC)			Metagenomic sequencing	*Streptococcus* equinus, Scardovia odontolytica and *Clostridium* hylemonae re significantly associated with the resistance of LARC patients to neoadjuvant chemoradiotherapy (nCRT)	Midha et al. (2023)
2021	CRC	*Fusobacterium* *Peptostreptococcus* *Campylobacter* *Streptococcus* *Treponema* Selenomonas *Enterococcus* Veillonella Leptotrichia Parvimonas Gemella Porphyromonas	*Bacteroides* *Clostridium* Prevotella Faecalibacterium	16S rRNA	The development of CRC may be partially driven by the symbiotic microbiota through DNA damage or abnormal signaling pathways	Huang et al. (2023)
2021	Gastrointestinal cancer	Capnocytophaga and *Helicobacter* (Upper gastrointestinal tumors) Porphyromonas (Rectum Adenocarcinoma)	----	PLS-DA	The microbial characteristics of gastrointestinal cancer exhibit significant organ specificity, with distinct differences in the microbial communities of upper and lower digestive tract tumors	Gutiérrez-Salmerón et al. (2021)
2021	microsatellite instability-high CRC	*Fusobacterium* nucleatum		quantitative PCR	*Fusobacterium* nucleatum may influence the progression of MSI-high CRC by promoting tumor-associated immune responses	Jiang et al. (2023)
2018	CRC	Bifidobacterium Fusobacterium nucleatum		quantitative PCR	The content of bifidobacteria is significantly correlated with the proportion of signet-ring cells in CRC.	Scott et al. (2020)
2024	Metastatic colorectal cancer (mCRC)	*Helicobacter* cinaedi Sphingobium herbicidovorans	---	RNA-SEQ 16S rRNA	The high expression of *Helicobacter* cinaedi and Sphingobium herbicidovorans is associated with poor prognosis in mCRC.	Sikavi et al. (2021)

### 2.2 Impact of intratumoral microbiota on the tumor immune microenvironment in colorectal cancer

#### 2.2.1 Recruitment and activation of immune cells

In CRC, the microbiota influences the immune environment of tumors by regulating immune cell recruitment, function, and the expression of immune checkpoint. For instance, as a typical pro-cancer bacterium in CRC, its abundance is significantly correlated with the polarization of tumor-associated macrophages (TAMs) and the methylation of the CDKN2A gene (Park et al., 2017). The FadA protein from *Fusobacterium* nucleatum activates the E-cadherin/β-catenin signaling pathway. This promotes IL-8 secretion, which leads to the formation of neutrophil extracellular traps (NETs) and inhibits the infiltration of CD8^+^ T cell (Rubinstein et al., 2013). Additionally, *Fusobacterium* nucleatum is closely related to immune evasion of tumor cells. By regulating immune cells function, these bacteria suppress anti-tumor immune responses, which in turn promotes tumor growth and metastasis (Hong et al., 2024; Jin et al., 2024). For example, *Fusobacterium* nucleatum can upregulate PD-L1 expression, directly inhibiting T cell activity, leading to immune evasion and affecting CRC efficacy (Gao et al., 2021; Gao et al., 2023). Furthermore, succinate produced by Prevotella spp. in CRC can weaken anti-tumor immune responses by binding to the SUCNR1 receptor. This binding inhibits mitochondrial oxidative phosphorylation (OXPHOS) and reduces IFN-γ secretion (Franke and Deppenmeier, 2018). Furthermore, the diversity of intratumoral microbiota may also influence the expression of immune checkpoints, impacting the effectiveness of immunotherapy. For instance, the enrichment of Proteobacteria in CRC can activate the TLR4 signaling pathway, inducing PD-L1 expression and leading to resistance to immunotherapy (Gopalakrishnan et al., 2018). Therefore, intratumoral microbiota not only affects the recruitment of immune cells but may also influence the immune response in CRC by regulating the activity of immune cells.

#### 2.2.2 Production and regulation of cytokines

Intratumoral microbiota can regulate the secretion of important cytokines in TIME through metabolic products, pathogen-associated molecular patterns (PAMPs), and epigenetic modifications, thereby altering immune responses (Kyriazi et al., 2024). For example, intratumoral microbiota can stimulate intestinal epithelial cells and immune cells to produce various cytokines such as IL-6, TNF-α, and IFN-γ, driving chronic inflammatory diseases and accelerating tumor progression (Li et al., 2022). An imbalance in intratumoral microbiota can increase immunosuppressive factors, which help tumors evade the immune system (Azevedo et al., 2020; Pratap Singh et al., 2023). Certain microbial metabolites can influence the polarization state of tumor-associated macrophages, which in turn affect the cytokines they secrete and alter the immune status of the tumor microenvironment. For instance, Prevotella has been identified in CRC liver metastatic tissues (Gu et al., 2024). Therefore, intratumoral microbiota influences the immune microenvironment of tumors by recruiting immune cells. Additionally, it shapes the immune characteristics of the tumor microenvironment by regulating the production and release of cytokines.

### 2.3 Metabolic pathways and immune regulation mediated by intratumoral microbiota in colorectal cancer

#### 2.3.1 Impact of intratumoral microbiota on colorectal cancer cell metabolism

Intratumoral microbiota in CRC not only affects immune evasion but can also alter the metabolic pathways of tumor cells by producing specific metabolites. For example, lactic acid, a common metabolic product of various bacteria, is often found in many tumors, including CRC. The use of lactate dehydrogenase inhibitors can significantly reduce tumor volume (Xue et al., 2023). *Fusobacterium* nucleatum can promote tumor cell glycolysis by activating HIF-1α and inhibiting OXPHOS, thereby providing energy and biosynthetic precursors for rapid tumor cell proliferation (Dipalma and Blattman, 2023; Midha et al., 2023). Additionally, Bullman et al. confirmed that *Fusobacterium* nucleatum promotes lipid synthesis in CRC by upregulating FASN (Mager et al., 2020). Furthermore, changes in the composition of intratumoral microbiota may lead to the reprogramming of host metabolic pathways, thereby affecting tumor growth and development. For instance, *Bacteroides* can promote CRC cell proliferation through the bile acid-FXR axis (Seki et al., 2021). The absence of beneficial bacteria, such as Bifidobacterium, is associated with tumor progression, while the enrichment of pathogenic bacteria, such as *Fusobacterium*, is closely related to malignant tumor progression (Long et al., 2024). Moreover, intratumoral microbiota can significantly impact targeted therapies for colorectal cancer by influencing host metabolic processes. For example, Bifidobacterium can enhance the efficacy of anti-PD-1 therapy through the inosine-ADORA2A pathway (Mager et al., 2020). Therefore, intervention strategies targeting intratumoral microbiota, such as using probiotics or altering dietary structures, may become new methods to improve CRC treatment outcomes (Sepich-Poore et al., 2021).

#### 2.3.2 Effects of metabolites from intratumoral microbiota on immune cells

Intratumoral microbiota in CRC significantly affects the function of immune cells and the tumor microenvironment through the production of various metabolites. Bacterial metabolites primarily consist of short-chain fatty acids (SCFAs) like acetate, propionate, and butyrate, along with amino acids and vitamins. These compounds can regulate the proliferation, differentiation, and function of immune cells, thus impacting tumor immunity (Scott et al., 2020). For instance, a novel oral hydrogel Oxa@HMI can generate SCFAs, regulate the intratumoral microbiota, stimulate various immune cells to initiate an immune response, and improve the efficacy of CRC treatment (Li et al., 2024). Additionally, butyrate enhances Treg function through HDAC inhibition, which promotes immune tolerance. Meanwhile, propionate activates IL-12 secretion in dendritic cell via GPR43 (Liu C. et al., 2024). Furthermore, SCFAs can regulate the activity of immune cells by activating G protein-coupled receptors (such as GPR41 and GPR43), promoting anti-tumor immune responses (Shi et al., 2023). In CRC patients, the reduction of SCFAs is closely related to tumor progression and immunosuppressive states, indicating that SCFAs may serve as potential biomarkers or therapeutic targets (Jin et al., 2024). Therefore, regulating gut microbiota to increase SCFA production holds promise for improving immune responses and treatment outcomes in CRC patients.

#### 2.3.3 Emerging evidence: Akkermansia muciniphila modulates antitumor immunity via metabolite-driven pathways

Recent advances highlight Akkermansia muciniphila (A. muciniphila) as a dual-functional modulator of colorectal cancer (CRC) through metabolite-immune crosstalk (Kropp et al., 2024; Zhu et al., 2024). As a mucin-degrading bacterium, A. muciniphila produces immunoregulatory short-chain fatty acids (SCFAs; e.g., propionate and acetate) that enhance intestinal barrier integrity and directly activate antitumor immunity (Lin et al., 2025). Propionate engages GPR43 on dendritic cells to amplify antigen presentation, driving CD8^+^T cell infiltration and IFN-γ production—critical for immune checkpoint inhibitor (ICI) efficacy (Gubernatorova et al., 2023; Li et al., 2025; Wang et al., 2023; Zhu et al., 2024). Concurrently, its surface protein Amuc_1100 activates TLR2/4 pathways, synergizing with SCFAs to suppress PD-L1 expression and bolster T cell-mediated tumor clearance (Wu et al., 2025). Paradoxically, A. muciniphila’s mucolytic activity may exacerbate inflammation in colitis-associated CRC contexts, underscoring context-dependent roles (Zhu et al., 2024). Crucially, elevated A. muciniphila abundance correlates with improved ICI responsiveness and survival in CRC patients, positioning it as both a predictive biomarker and a next-generation probiotic candidate for microbiota-directed therapies (Ni et al., 2022).

### 2.4 Intratumoral microbiota promotes colorectal cancer metastasis: mechanisms and pathways

Tumor-resident microbiota critically drive colorectal cancer metastasis through coordinated mechanisms targeting cellular plasticity and microenvironment remodeling. For example, *Fusobacterium* nucleatum induces epithelial-mesenchymal transition (EMT) by activating the PI3K/AKT signaling pathway, thereby enhancing the migration and invasion capabilities of tumor cells (Fan et al., 2024). Concurrently, microbial metabolites such as bile acids engage FXR/TGR5 receptors to induce IL-1β secretion by hepatic macrophages, disrupting endothelial barriers and priming pre-metastatic niches in distant organs (Liu L. et al., 2024). This spatial reprogramming is further amplified by LPS-stimulated neutrophil extracellular traps (NETs), which entrap circulating tumor cells to facilitate vascular adhesion and extravasation (Kyriazi et al., 2024). Notably, microbial communities exhibit context-dependent influences: For example, butyrate has been shown to inhibit the proliferation of tumor cells and induce apoptosis by regulating gene expression through the inhibition of histone deacetylases (HDAC), affecting the growth and migration of tumor cells (Kim et al., 2020). However, in certain cases, butyrate can promote the activation of signaling pathways related to metastasis in the tumor microenvironment (Kong et al., 2022; Zou et al., 2024). These findings establish intratumoral microbiota as master regulators of the metastatic cascade, offering mechanistic links to clinical treatment failures.

## 3 Role of intratumoral microbiota in colorectal cancer treatment

### 3.1 Impact of intratumoral microbiota on sensitivity to chemotherapy and radiotherapy in CRC

The composition of intratumoral microbiota can significantly influence the response of CRC to chemotherapy and radiotherapy. For example, *Fusobacterium* nucleatum is associated with chemotherapy resistance in CRC, possibly due to its promotion of immunosuppressive effects in the tumor microenvironment (Bullman, 2023). Additionally, the diversity of intratumoral microbiota in CRC is closely related to patient prognosis, with low-diversity microbiota typically associated with poorer treatment responses and survival rates (Gopalakrishnan et al., 2018). RNA sequencing of tissues from 105 rectal cancer patients identified 12 microbes linked to treatment response. This finding confirms that the bacterial composition in these tumors significantly differs based on treatment outcomes and serves as an independent biomarker for predicting responses to neoadjuvant chemotherapy and radiotherapy (nCRT) (Sun et al., 2023). Furthermore, a metagenomic sequencing study involving tissues from 73 patients with advanced rectal cancer found that key bacteria, such as *Streptococcus*, *Fusobacterium*, and *Clostridium*, are strongly linked to resistance against nCRT. This study also revealed that butyrate derived from the microbiome may play a role in reducing nCRT efficacy (Huang et al., 2023). In terms of radiotherapy, significant changes in the composition of intratumoral microbiota occur in patients undergoing radiotherapy, which may be related to the efficacy of radiotherapy (Zhang J. et al., 2024). For instance, some studies have found that *Fusobacterium* nucleatum and *Fusobacterium* canifelinum are enriched in hypoxic CRC and are closely related to patient prognosis, suggesting potential interactions between hypoxia, microbiota, and radiotherapy responses (Benej et al., 2024). Therefore, interventions targeting intratumoral microbiota may become a potential strategy to enhance CRC patients’ sensitivity to chemotherapy and radiotherapy (Cogdill et al., 2018).

### 3.2 Role of intratumoral microbiota in colorectal cancer immunotherapy

Intratumoral microbiota critically regulates immunotherapy responses by altering the TIME and immune checkpoint pathways. For instance, the purple photosynthetic bacterium Rhodopseudomonas palustris (RP) has a symbiotic relationship with the anaerobic bacteria *Proteus mirabilis* (PM) in tumors. This microbial symbiont, known as A-gyo, not only eliminates various tumors, including colorectal cancer (CRC), but also induces durable anti-tumor immunity, offering long-term specific protection (Goto et al., 2023). Moreover, *Lactobacillus* rhamnosus GG enhances CD8^+^ T cell responses in the gut, which is essential for strengthening anti-tumor immune responses (Owens et al., 2021). Furthermore, the diversity of intratumoral microbiota and the abundance of specific microbes significantly impact the efficacy of immune checkpoint inhibitors (ICIs). Low-diversity microbiota may contribute to immune suppression and diminish the effectiveness of immunotherapy (Farhadi Rad et al., 2024; Jiang et al., 2023). For instance, the abundance of *Fusobacterium* nucleatum is associated with tumor immune evasion mechanisms, potentially leading to resistance to ICIs (He et al., 2022). For patients with CRC liver metastasis, RIG-I lactylation inhibitors target *E. coli* in tumor tissues. This action suppresses M2 macrophage polarization and increases patient sensitivity to 5-fluorouracil (5-FU) (Gu et al., 2024). A study found that certain combinations of intratumoral microbiota can predict how patient respond to ICIs. This indicats that the composition of microbiota may serve as a potential biomarker for assessing treatment responses and prognosis PMID: 39439901. Modulating the composition of intratumoral microbiota could improve the efficacy of immunotherapy and potentially lead to better prognoses for patients (Shen et al., 2021a). Therefore, understanding the interactions between intratumoral microbiota and immunotherapy can help develop personalized treatment strategies to enhance outcomes for CRC patients.

## 4 Potential of intratumoral microbiota as diagnostic and prognostic biomarkers in colorectal cancer

### 4.1 Role of intratumoral microbiota in early diagnosis of colorectal cancer

Changes in intratumoral microbiota are strongly linked to the development of CRC, especially in its early stages. Studies comparing the microbiomes of tumor tissues to those of nearby normal tissues have found that some microbes are significantly more abundant in tumor tissues. For example, *Fusobacterium* nucleatum and *Bacteroides fragilis* have been shown to be associated with the malignancy of CRC and patient survival rates (Kunzmann et al., 2019; Mondal et al., 2025; Shen X. et al., 2021). Furthermore, microbiome detection in the blood of patients with stage II/III-IV CRC has shown that the detection rates of *Escherichia coli*, *B. fragilis*, and *Candida* albicans are significantly related to CRC liver metastasis and overall survival rates, emphasizing the prognostic value of these microbial biomarkers (Zhang Q. et al., 2024). Another study conducted quantitative PCR testing on tumor tissue samples from 1,313 CRC patients. It found that Bifidobacterium DNA was present in 30% of these samples, and its content was significantly related to the proportion of signet-ring cells in tumors. This suggests that Bifidobacterium could be an indicator of specific colorectal cancer characteristics (Kosumi et al., 2018). Additionally, liquid biopsy techniques based on intratumoral microbiota are becoming a promising non-invasive detection method that can assist in the early diagnosis of colorectal cancer (Ramos et al., 2023; Raza et al., 2022).

### 4.2 Role of intratumoral microbiota in predicting treatment responses and prognosis in colorectal cancer

Intratumoral microbiota shows significant potential in predicting treatment responses and prognosis in CRC. For example, the levels of intratumoral microbiota are associated with the number of tumor-infiltrating T cells, indicating that these microbes might influence treatment outcomes by altering immune responses (Luu et al., 2023; Pratap Singh et al., 2023). Additionally, the diversity of intratumoral microbiota is positively correlated with patient survival rates, indicating that changes in microbial abundance may serve as a new tool for predicting prognosis in CRC patients. Prospective cohort studies suggest that a dietary pattern known as the ‘sulfur microbiome diet’ may be associated with specific CRC molecular subtypes or the presence of intratumoral microbiota. Long-term adherence to the sulfur microbiome diet may be related to high expression of prostaglandin synthase 2 (PTGS2) and increased risk of distal CRC with Bifidobacterium negativity (Sikavi et al., 2021). These findings provide new insights into the relationship between diet, intratumoral microbiota, and CRC, suggesting that dietary modulation may serve as a preventive strategy for high-risk populations.

## 5 Challenges and future research directions

### 5.1 Technical challenges in intratumoral microbiome research

Research on intratumoral microbiomes encounters several technical challenges, mainly due to low microbial biomass and the inability to certain microbial species. These challenges complicate and hinder in-depth studies of tumor microbiota. Current research methods often have difficulty accurately capturing the diversity and changes in tumor microbiota, especially in different tumor microenvironments. To overcome these technical barriers, researchers are developing new methods with high spatial and temporal resolution, such as the combined application of multi-omics technologies (including genomics, transcriptomics, proteomics, and metabolomics). For example, comprehensive profiling of microbial communities in tumor samples through next-generation sequencing (NGS) can obtain complete lineages of microbes without the need for culturing and determine the composition and abundance of microbes in different digestive system tumors (Xuan et al., 2024). Other studies have confirmed strong correlations between *Helicobacter* cinaedi and Sphingobium herbicidovorans in metastatic colorectal cancer tissues with specific genes (such as SELENBP1 and SNORA38), providing new ideas for personalized treatment (Feng et al., 2024). These technological advancements will help to better understand the interactions between tumor microbiota and the tumor microenvironment, thereby providing new ideas and strategies for early diagnosis and treatment of cancer.

### 5.2 Ethical and safety issues in clinical applications

When translating the research findings of intratumoral microbiomes into clinical applications, ethical and safety issues cannot be overlooked. First, interventions in the microbiome could have unexpected effects on patient health, necessitating in-depth discussions of related ethical issues. For example, we need to address how to ensure patients receive adequate informed consent for microbiome interventions and how to assess and monitor the safety of these interventions. Additionally, individual differences in microbiomes make it challenging to create universal treatment plans (Liu et al., 2021). Therefore, future research should focus on developing personalized microbiome modulation treatment plans while ensuring ethical considerations to maintain patient safety and treatment effectiveness.

### 5.3 Heterogeneity in sample collection of intratumoral microbiota

There is significant heterogeneity in the collection of intratumoral microbiota samples, posing challenges to the reproducibility and reliability of research results. Various factors, including patients’ tumor microenvironments, treatment histories, and lifestyles, can influence the composition and function of microbiota (Zhang et al., 2020). For instance, CRC-related pathogenic bacteria (such as *Clostridium*, *Bacteroides*, Eubacterium, and Prevotella) exhibit high variability across different samples (Barc et al., 2008; Benjamin et al., 2012). Therefore, standardized protocols for collecting and processing samples must be established to reduce variability in microbiome studies. Additionally, specific sample collection strategies may need to be developed for different types of tumors to ensure that the true state of tumor microbiota can be captured. Future research should focus on the heterogeneity of samples and validate the relationships between microbiomes and tumor characteristics. This can be achieved through large-scale, multi-center clinical trials, which will enhance the application of microbiome research in cancer treatment.

## 6 Conclusion

**FIGURE 1:**
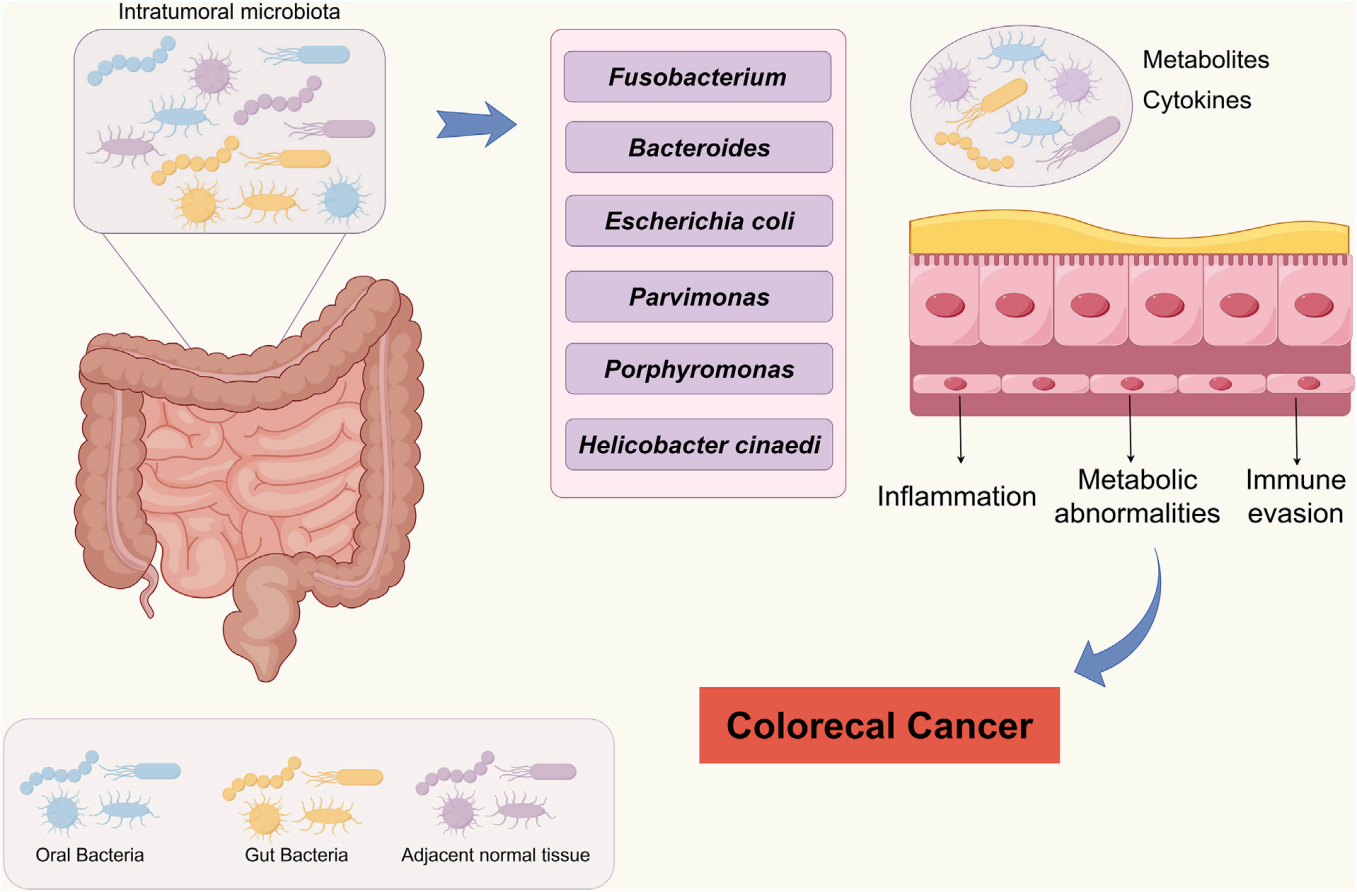
Schematic representation of intratumoral microbiota driving colorectal carcinogenesis.

This review reveals the mechanisms by which the intratumoral microbiome affects treatment response through a metabolic-immune regulatory network in CRC (Figure 1). This not only provides new biomarkers for predicting CRC progression but also offers potential new targets for cancer therapy. However, current research on intratumoral microbiota still has some controversies and gaps, and how to effectively integrate these discoveries into clinical applications is a pressing issue. Future studies should systematically explore the characteristics of microbial communities in different patient populations, tumor types, and treatment regimens, using big data and AI to pull out valuable insights and promote the progress of clinical translational research. In clinical practice, focusing on the impact of intratumoral microbiota could lead to new treatment strategies. By adjusting microbial communities, we hope to improve patients’ response rates to chemotherapy or immunotherapy, but we need to rigorously validate the effectiveness and safety. In summary, intratumoral microbiota have great potential in the research and development of CRC. Encouraging collaboration across disciplines can help us better understand their role in tumor biology, bringing new hope and treatment options to patients.
